# Hospital and Institutional News

**Published:** 1913-11-01

**Authors:** 


### HOSPITAL AND INSTITUTIONAL NEWS.

### THE FUTURE MANAGEMENT OF YORK HOSPITAL.

The long-delayed report of the Special Committee appointed by the governors of York County Hospital to consider Sir Cooper Perry's Report has been issued at last. It makes virtually only one recommendation, and for the rest is an attempt to explain away everything in the Report which indicated mismanagement. To be precise, the most crucial instance of this is the Special Committee's attitude to the mortuary case. It has been admittedly proved that a patient was taken to the mortuary while still alive, but the Committee state that the residents' charge is disposed of on the ground that they were equally responsible for it. This "You're another" attitude will hardly impress the public with the genuineness of the hospital's desire for reform. The fact was proved, and new regulations have been necessary. It is these new regulations on which it would have been more becoming to enlarge. The attempt to veil facts under similar recriminations, we fear, runs through the whole document. But it is the facts which are important, and with these alone the public is concerned. With regard to the strained relations between the residents and the Matron, the Committee agrees that the hospital must suffer under such conditions, but again runs off by erroneously remarking that these conditions are "very commonly found to exist." They quote Dr. Macdonald's and Dr. Gayner's dictum that "the Matron is always difficult to approach" and their following recommendation for "a little more suavity," and then state their opinion that she "has overworked herself," and recommend that she shall "have full authority to engage sisters," and so forth. Beside these generalisations a fact again is worth much. The fact is this. Last week the nursing papers contained an advertisement to the effect that at York County Hospital a lady superintendent is required. This change in the *personnel* will be noted by the public. The single clear-cut recommendation is that "during the intervals between the meetings of the house committee the ultimate control of the hospital shall be vested in a member of the house committee, to be nominated annually by the committee, and a member of the medical board to be nominated annually by that board." In other words, this single recommendation is one which, without discussing the merits of the dual control involved, is the very same in principle as that suggested in our Report on the institution nearly two years ago. There the need for "the introduction of a controlling hand" was insisted on as the most necessary and fundamental reform. Whether the public of York, which was healthily awake in September to the need for knowing precisely what immediate steps were being taken, will be satisfied with this report remains to be seen. What the facts are, apart from the verbiage, we have done our best to indicate.

### WHAT IS THE LOOSE SCREW AT YORK?

Well-informed and trusted correspondents, who are familiar with the inner working of York County Hospital, declare that the cause of all the trouble at York was mainly the fact that the House Committee had for its Chairman an esteemed divine, who is eighty-five years of age, and has been completely deaf for very many years. Further, that the control has been practically in the hands of one of the honorary staff and a former Matron, who is married to one of the staff, and is a most active member of the House Committee. We publish these opinions for what they may be worth, because we feel that in view of the character of the report of the Special Committee it is essential in the public interest that an opportunity should be given to the York authorities to deny or confirm these statements in the interests of the patients, the public, and all concerned. It is lamentable that the Special Committee has not acted upon the recommendation to increase the staff, and so abolish the pernicious and costly system altogether of introducing special nurses whenever a heavy case has to be provided for. A little more frankness, the exhibition of some courage, and the realisation of the position which the York County Hospital holds at present in the opinion of the hospital world would surely cause a franker and more statesmanlike position to be taken up by those who are at present responsible for the administration of the affairs of York County Hospital.

### SALARIES IN LONDON COUNTY MENTAL HOSPITALS.

The Asylums Committee of the London County Council again offer official acknowledgment of the fact that resident medical officers in mental hospitals are increasingly harder to secure. The Committee, therefore, proposes that a higher scale of salaries should be adopted representing just over 14 per cent., and that in five years' time, should the staff be unchanged from its present position, a maximum increase of some 25 per cent. should be adopted. The proposed rise in salaries represents an immediate increase of £1,620. Recent articles in The Hospital have shown, however, that, though salaries may be improved, that is not the only change required to remove the burdens of boredom and the actual disadvantages now oppressing the assistant medical officers of our mental hospitals throughout the country. The isolated position of the assistant medical officer, his temptations to fall into the merest routineer, the absence of properly co-ordinated opportunities for research, his disability for marrying, and the small number of superintendentships available all tend to make mental work unattractive at the present time to all but the keenest enthusiasts. These causes are admittedly difficult to remedy, and probably impossible to remedy at once. That is why the proposed increase in salaries, with the immediate improvement that it offers, is to be welcomed, and will be appreciated, as far as it goes.

### THE REVISED SALARIES IN DETAIL.

The present scale of remuneration for assistant medical officers does not provide an annual increment for any grade except the first. The fifth and sixth assistants receive £170 a year each, the fourth £190 a year, the third £210 a year, and the second £250 a year; in each case with board, lodging, and washing. These emoluments are valued at £90 a year. The first assistant receives an annual salary of £310 a year, rising by £25 a year to £410, with the same residential emoluments. The Asylums Committee of the London County Council is now revising the salary scale as follows:—

| | Commencing salary yearly | Annual increment yearly | Maximum salary yearly |
|---|---|---|---|
| First A.M.O. ... ... ... | £350 | £25 | £450 |
| Second A.M.O. ... ... ... | 290 | 10 | 330 |
| Third A.M.O. ... ... ... | 250 | 10 | 280 |
| Fourth A.M.O. ... ... ... | 220 | 10 | 250 |
| Fifth A.M.O. and all appointments under ... ... | 200 | 10 | 220 |

With board, lodging, and washing in each case.

### RAILWAY DISASTERS AND THE HOSPITALS.

Mr. Allen Naldrett, secretary-superintendent of the Royal Southern Hospital, Liverpool, compiled an interesting report on the St. James's Railway disaster, which is a valuable record of hospital emergency work. "I beg to report," he writes, "that on Wednesday, October 15, in response to a telephone call received at 2.45 p.m., the ambulance proceeded at 2.50 p.m., attended by Dr. Findlay, to St. James's Station. The message having reported a collision, immediate action was taken to cope with a large number of injured. The honorary medical officers were summoned and the services of doctors were requisitioned from other hospitals in case of need. All the couches from the Out-Patient Department were removed to the Killick Wards, and the old office was also utilised as a ward with three couches. The Matron had meantime drawn from the home and the wards a large staff of nurses, and on the arrival of the first patient from the disaster everything was in readiness. Altogether twenty-five persons were brought in, in quick succession, all of whom were dealt with by members of our own honorary and resident medical staffs and nurses. Of the twenty-five persons, one died just after admission, and two were well enough to proceed to their homes the same evening. Of those remaining, there were English: males 4, females 4; Foreign: males 13, females 1; total 22. Ten of the patients have since been discharged, and the twelve patients remaining under treatment are progressing favourably, although one of them is so seriously injured that his final recovery is doubtful. The various Consuls, Railway and Shipping representatives, and others interested in the injured have been given every facility to prosecute their inquiries and to assist the patients. Much credit is due to the honoraries for the ready manner in which they responded to the summons and for the supervisory and operative work they so promptly performed. The work of the members of the resident medical staff, both at the scene of the disaster and at the hospital, cannot be too highly commended, nor can that of the Matron and nursing staff for the disciplined assistance so quickly and ably rendered. The commendation of the Committee is due to the porters, engineering and clerical staffs for the respective emergency duties they performed upon the occasion. The Committee's thanks are also due to the medical officers of other institutions, who so willingly attended to give their services in case of need, and to several members of the public, who assisted in the removal of couches and the admission of patients."

### THE PUBLICATION OF EMERGENCY REPORTS.

We have often argued that every emergency in which a hospital has to take part, whether it be in a fire within the institution, or in an accident such as this, should be promptly followed by a published report giving the lessons which have been learnt from it. We therefore cordially welcome the above as a beginning. But our readers will agree that the report would have gained by further detail in respect of the actual difficulties experienced in giving the aid which is here recorded, and for which so many words of thanks are rightly expressed. The gratifying statement that "immediate action was taken to cope with a large number of injured" must have depended on an efficient administration. If all ran smoothly, as we have no reason at all to doubt, the method adopted becomes highly important and would have made instructive reading to all hospital managers.

### MR. A. CHAMBERLAIN AND TROPICAL MEDICINE.

The great feature of the annual dinner of the London School of Tropical Medicine last week was the account of the allocation of the recently collected special funds. Dr. F. M. Sandwith presided and Mr. Austen Chamberlain, who was the principal guest, explained that of the £100,000 asked for some £70,000 had been obtained. Acting on the advice of the committee of management of the School and of the board of the Seamen's Hospital, £15,000 had been allocated to a not very large but essential extension of the school buildings; secondly, a fund for research had been started, thanks to Sir W. Bennett's allocation of Lord Wandsworth's bequest of £10,000; and, thirdly, an endowment producing an annual income of about £1,400 had been provided for the School. Lastly, through the co-operation of the Seamen's Hospital, arrangements had been made for providing those men who returned from their work suffering from a tropical disease, but without the means for securing the best special knowledge and treatment, with these two things in such a way as not to embarrass the recipients. In reply, Dr. Sandwith referred to that part of the endowment scheme which involved the establishment of special beds for special individuals, and stated that he was glad to add that one of the hospital wards in future was to be known as the Chamberlain Ward. The cheers that greeted this statement and the announcement that the School is now fairly flourishing in funds and most flourishing in membership must have well rewarded Mr. Chamberlain for his labours. Portraits of Sir Patrick Manson were presented by Mr. James Cantlie and Dr. W. T. Prout on behalf of the subscribers, and a new sphere of usefulness and importance in the history of the School and its work has happily begun.

### PANEL DOCTOR AND CRIMINAL LIBEL.

Dr. Horace Dimock, who went to Wisbech some months ago to complete the tale of panel doctors under the Insurance Act, was charged at Wisbech last week with criminal libel. The information was laid by Dr. Henry Meacock, of Wisbech, who is chairman of the Isle of Ely branch of the British Medical Association. The solicitor for the prosecution alleged that the libel had reference to the sending and publishing of a postal packet containing a postcard with libellous matter printed upon it. On being arrested the defendant said, "I wrote no letter." He was remanded on bail for a week, two of his colleagues on the panel standing as sureties. Among Dr. Dimock's previous appointments was that of senior house surgeon to Addenbrooke's Hospital, Cambridge. He graduated in arts at Cambridge in 1903 and in medicine in 1907, and was once resident anæsthetist at St. George's Hospital. Since writing the above Dr. Dimock has been found dead in his room at Stretham, near Ely. Whether or not death was due to suicide remains to be seen.

### DEATH OF A GREAT FRENCH SURGEON.

Those who met Dr. Lucas-Championnière at the late International Congress of Medicine will have experienced a painful surprise and shock by the news of his sudden death. For he seemed as full of energy and health as a man could be, and would certainly have been suspected of about fifty-five years instead of the seventy which he had actually lived. At a luncheon given by the Editor of the *Lancet* at the Hyde Park Hotel, he spoke with all the graceful eloquence and fluency of the accomplished orator he was; and his spare, athletic frame did not look like that of a man for whom sudden collapse was a possibility. He was actually reading on October 22 to a private audience an address which he was to deliver in public the next day, when he suddenly fell and died before medical assistance could be obtained. Lucas-Championnière was a man and a surgeon of the highest eminence, whose activities covered many fields. His two most important contributions to progress were his enthusiastic acceptance of Listerian principles from the earliest days, and his advocacy of mobilisation and massage in the treatment of fractures. He went to Glasgow in 1868 and to Edinbugh in 1875 in order to study under Lister himself; and remained all his days an exponent of the exact technique as perfected by the great discoverer of antiseptic principles. His death is attributed to embolism; and French surgery is poorer by the loss of one of its most brilliant personalities and exponents.

### A PROFESSIONAL QUARREL.

A somewhat unusual professional quarrel between members of the medical profession has ended in a libel action at the Bedford Assizes. The plaintiff was a Dr. Herbert Sloman, who some few years ago got in touch with a Dr. Clark, of Bedford, with a view to buying the latter's practice. This he arranged to do, subject to a satisfactory report from an accountant. The report, however, did not confirm in every particular what Dr. Sloman had been told about the practice, so he did not complete the purchase. He did, however, decide to settle down in Bedford, and make a practice for himself by the process colloquially termed "squatting." Dr. Clark considered himself aggrieved by this, and brought the affair to the notice of the local British Medical Association as a breach of ethics. As a result of this complaint Dr. Sloman agreed that for three years he would refuse to attend any of Dr. Clark's patients. After the expiry of this period Dr. Clark was still malcontent, and certain members of the British Medical Association, at his instigation, passed a motion containing derogatory references to Dr. Sloman. Copies of this were circulated in halfpenny wrappers; hence the libel action. As a result of the Judge's suggestion, after the first day's hearing, the parties came to terms; and Dr. Sloman got a verdict and substantial damages.

### BRENTFORD GUARDIANS' REPLY TO CRITICISMS.

At a special meeting of the Brentford Board of Guardians last week a report was presented by the infirmary committee in reply to Colonel Carr-Calthrop's resolutions, already discussed in our columns. The report traverses his allegations and can perhaps be best summarised by the following sentence extracted from it: "The committee would point out that no numbers have been fixed for the wards, and that no strictures as to overcrowding have been passed upon the Guardians by the Local Government Board inspectors." But, as we said before, Local Government Board inspection is well known to be inadequate. Dr. Williams said that the Colonel's view was that the severe operation cases should be segregated into one of the smaller wards, but the medical men's views were that these wards were less suitable than the bigger ones. An infirmary could not be likened to an ordinary general hospital. At the infirmary all cases had to be taken as they came. He denied that there was a need for segregation of all different forms of disease. Colonel Carr-Calthrop said he was glad to see that the committee had done what he wished as to identification of the wards, and he agreed that the Local Government Board should be asked to notify the accommodation. As to the rest of his complaints, he should await the further report with interest. The report was agreed to, but reform is still required.

### THE ROYAL COMMISSION ON SYPHILIS.

The *personnel* of the Royal Commission to inquire into the subject of venereal disease is now announced. Lord Sydenham is chairman, and its members are Sir David Brynmor Jones, K.C.,

M.P.; Mr. Philip Snowden; Sir Kenelm Digby, formerly Permanent Under-Secretary at the Home Office; Sir Almeric FitzRoy, Clerk to the Privy Council, who was chairman of the Departmental Committee on the Midwives Act in 1909; Sir Malcolm Morris, F.R.C.S.; Sir John Collie, M.D.; Mr. Arthur Newsholme, C.B., Chief Medical Officer to the Board of Education; Canon Horsley; the Rev. J. Scott Lidgett; Mr. F. W. Mott, F.R.S., M.D.; Mr. J. E. Lane, F.R.C.S., senior surgeon at St. Mary's and the London Lock Hospital; Mrs. Scharlieb, M.D., consulting gynæcologist to the Royal Free Hospital; Mrs. Creighton; and Mrs. Burgwin. The Secretary to the Commission is Mr. E. R. Forbes, of the Local Government Board. The terms of reference are to inquire into the "prevalence, effects, and means of alleviation of venereal diseases in the United Kingdom," it being understood that no return to the policy or provisions of the Contagious Diseases Acts of 1864, 1866, 1869 is to be regarded as falling within the scope of the inquiry."

### THE STATUS OF THE WORKHOUSE CHAPLAIN.

Everyone will regret the circumstances under which the King's cheque for five guineas, sent on request of the Chaplain of Newmarket Workhouse for assistance in holding an annual treat, has been deprived of its object, since the Newmarket Guardians have decided that the treat shall not be held. Last year the treat was discontinued, and the mistake of the Workhouse Chaplain, who was appointed last Whitsun, consisted in forgetting this, for on receiving from his predecessor the balance of the treat account, with the King's name at the head of the list of subscribers, he applied to Lord Stamfordham for the subscription, usual till last year, when it was not applied for. This was at once received, when the Chairman of the Board of Guardians took occasion to remark that the application for a subscription was not in order, since there was a resolution on the minutes stating that no official should solicit subscriptions without the special direction of the Board. The Chaplain, thus challenged, replied that he was aware of this resolution, but he considered that it did not apply to himself, but only to the resident officers of the workhouse. On the matter being referred to the house committee, it was recommended that the treat should not be held, and the cheque, consequently left in the hands of the Chaplain, who has since written to Lord Stamfordham for instructions as to its disposal. Clearly, the Guardians have placed their Chaplain in a difficult position, in which, however, the sympathy of his institutional colleagues will go out to him. But in leaving the cheque in the Chaplain's hands not only the Chaplain is repudiated. Moreover, the very important question of the status of the Workhouse Chaplain is now raised, and that status may gain from clearer definition, as it is one which must be safeguarded in the best interests of Poor-Law institutional life and work. Local feeling is strongly in favour of the treat being held this year at any rate, and this seems the best solution of a difficulty which was exacerbated by the Guardians' want of tact. What do our readers think?

### DEGREES OF ILLNESS AMONGST SICK PAUPERS.

As the result of inquiries from a correspondent, following on some comments in The Hospital, the Local Government Board have given some valuable new statistics in Part I. of their Report for 1912-13, which has just been issued. Thus the classification of sick paupers, according to their degree of illness, has been dealt with, this being a point on which the absence of reliable information has hitherto been a serious drawback to the student of hospital questions, more especially in reference to the value of Poor-Law infirmaries for clinical teaching. The total number of invalid paupers on a certain date is given as 97,926. Of these 26,930 (Class B) may be ignored as cases "under medical care, but not needing active or continuous treatment, or special nursing." Of the remaining 71,000 the number of acute cases is given at 22,329, or 31 per cent., and the number of chronic cases at 48,667, or 69 per cent. The chronics are defined as "those who were undergoing no active change, or who had been, or were likely to be, for six months under medical treatment." All the remainder of Class A were called acute. The 22,329 acute cases were: medical as to 14,970, and surgical as to 7,359. The 48,667 chronic cases were: medical as to 39,869, and surgical as to 8,798. All these figures relate to indoor paupers, but similar statistics are given with regard to recipients of outdoor medical relief. Amongst other new and interesting figures given in the Report are those relating to the classification of sick paupers as between the ordinary workhouses, the sick wards, and the separate infirmaries. It is remarkable to find that of the total number of indoor sick paupers in England and Wales (97,926), 47,525, or practically half, were being treated in the sick wards of the workhouse proper, and not in separate infirmaries.

### THE LATE MR. T. F. MYERS.

The death of Mr. T. Frederick Myers removes a personality well known and well loved amongst hospital workers. For sixteen years Mr. Myers was secretary of the Yarrow Convalescent Home, and, although at the time of his death not actively engaged in the work of that institution, he was a keen and prominent member of the Incorporated Association of Hospital Officers to the end. The deceased gentleman was an old City of London School boy, and was very well known in commercial circles. He attained the age of seventy nearly four years ago, when he retired from the Yarrow Convalescent Home, being succeeded by the present secretary, Mr. W. J. Sheldrick. Mr. Myers, however, was unable to restrain his energies, and he took up, soon after, a position with one of the great City Companies. He was never married, and was a particularly amiable and unselfish man, ready at any time to go to great personal inconvenience for the benefit of others.

The funeral took place at Hampstead on Thursday, last week, many members of the Incorporated Association of Hospital Officers being present. Mr. Myers was a vice-president, and at the time of his death vice-chairman of this body.

### A LONDON HOSPITAL IN THE MARKET.

In order that the governors may devote their whole attention to the Northwood Institution, Mount Vernon Hospital, Hampstead, is now for sale. It is not often that a hospital thus comes under the hammer, and a somewhat different sound is given to the familiar category of wards, administration and out-patient departments, extent of accommodation, and so on when these are brought forward, not to describe the scope of the work undertaken, but to attract a purchaser. What term, we wonder, will the crafty imagination of the agents devise for this abandoned hospital? Neither the adjectives "very desirable," "unique," nor the stock terms of description seem to suit; and yet, awaiting what allied or alien use are 120 beds, a nurses' home, and a new pathological laboratory? The site alone, which consists of nearly three acres, should be perhaps its most valuable commercial asset, and offers are understood to have been received.

### THE CRISIS AT CAMBERWELL INFIRMARY.

As we foretold at the time, the regulations passed some while ago allowing corporal punishment in certain cases to the authorities of Camberwell Infirmary are causing trouble to those who sanctioned them. Indeed, the matter is one of public importance since over 800 ratepayers of Camberwell have signed a petition praying the Local Government Board to hold an inquiry into the management of the infirmary. The petition alludes to the alleged flogging of child patients, and goes on to state that difficulties are put in the way of parents and friends who desire to visit the patients. Another complaint in the petition refers to the proposed increase in the salary of the Medical Superintendent, Dr. Keats, and states that numerous protests on these points have been made by the ratepayers to the Guardians without effect. The infirmary committee in the meantime has had its hands full by inquiring into certain complaints against Dr. Keats, which have been preferred against him by Dr. Riches, the assistant medical superintendent, Dr. Soutter, and Dr. Nairn alleging that they were debarred from social amusements and not consulted by him. Dr. Keats has also made charges of insubordination against them, although Dr. Riches at least was recently thanked by the committee for services rendered by him in the absence of his chief. The infirmary committee, however, has decided to ask the three medical men to resign. Any administration which has not succeeded in satisfying the outside public nor in satisfying a section of its own officers must have some serious defect; but, in view of Dr. Keats's known abilities and of the confused state of the interests involved in this regrettable condition of affairs, it is exceedingly difficult to fix upon the quarter primarily responsible. For this reason an impartial public inquiry is to be welcomed, and we hope that it will be held without delay.

### THE PROPOSED SANATORIUM NEAR WELLS.

At a recent meeting of the Tuberculosis Sub-Committee of the Somerset County Council it was decided by thirty-three votes to twenty-seven to acquire the site under Lyatt, near Wells, Somerset, for a county sanatorium for consumption. The Mayor of Wells, however, in objecting to the site chosen, said: "I am told by expert evidence that the marsh air of the moors, with the wind blowing as it does just on the site, makes it one of the worst sites that could possibly be chosen;" and Lord Hylton was of the opinion that it was unfair that Wells should have another institution of the kind in its midst. The Chairman of the Council, the Right Hon. Henry Hobhouse, said that the site had been approved by their medical experts, Dr. Savage and Dr. Short, and provisionally by the expert of the Local Government Board. Dr. Meredith, replying to the Mayor of Wells, expressed his opinion that there was no possible fear of infection. In Wells itself the inhabitants are much divided in opinion as to whether the erection of a sanatorium would be beneficial and add to the health and prosperity of the place or otherwise; and some of the residential landowners in the neighbourhood of the site object. The site consists of about sixty acres of land. A memorial is to be presented against the adoption of the site; but it has been chosen, as we have said, by the Tuberculosis Sub-Committee of the Somerset County Council, and we refer to the matter here mainly to give yet another instance of the extraordinary way in which the public imagines a properly conducted sanatorium to be a centre of infection. As to the landlords, we have previously given the arguments and experience which show that a new sanatorium tends to raise, not to lower, rents.

### THE FISH SUPPLY OF WANDSWORTH INFIRMARY.

The Wandsworth Board of Guardians are having considerable trouble with their fish supply. Unfortunately, when the tender was accepted the Board was warned against ratifying the lowest tender, but, contrary to advice, they agreed to adopt it. Only a few weeks passed when numerous consignments had to be condemned, and the staff at the infirmary was put to the inconvenience of having to go out of the institution and purchase fish in the open market. A proposal was submitted to the Board that the tenderer should be released from his contract, but the Guardians declined to accept this very easy way out of what was a great difficulty. Possibly the quality of the fish may improve, and for the sake of the patients and the staff it is to be hoped that it will. The incident is a warning against the yielding to the common temptation of Boards of Guardians to accept the lowest tenders, and as such should be an example and a warning.

### THE PROFESSION AND THREATENING LETTERS.

The case, already alluded to in our columns, in which a picture-framer was charged with sending to Dr. W. Jarvis Bell a threatening letter demanding money, ended in the conviction of the accused. Mr. Justice Lush remarked that a most serious and cowardly offence had been committed, and sentenced the prisoner to three years' penal servitude. The profession will congratulate Dr. Bell on having the prisoner brought to book, for this kind of attack is one to which medical men are particularly open by the nature of their work. We hope the verdict and sentence will not be lost on intending blackmailers.

### A SCHOOL CLINIC FOR ACTON.

After the abortive negotiations with the Acton Cottage Hospital and the West London Hospital, already discussed in our columns, for the treatment of school children suffering from adenoids and tonsils, the Acton Education Committee have now approved the provision of a school clinic for such treatment, which would seem to be the most satisfactory solution of the problem. The initial cost of fitting the clinic is estimated at £100, and for dental work an additional similar amount; and the yearly upkeep is expected to be £286. Suitable accommodation is to be sought from the Acton Council, probably at the Council Offices; and when this is obtained applications are to be invited for the appointments of dentist, surgeon, anæsthetist, nurse, and clerk.

### COMBINATION AMONG SCHOOL MEDICAL OFFICERS.

Rumours of the existence among school medical officers of dissatisfaction with the prospects of their particular branch of the Public Health Service have been current for some time. Apparently steps are now being taken to bring school medical officers into closer communication for the ventilation of grievances and for mutual support. A large number of school medical officers are members or fellows of the Society of Medical Officers of Health, and recently these officers, engaged solely or chiefly in school inspection work, have formed a group within the Society. The first meeting of this subsection will take place on November 7. There appears to be no doubt but that in many parts of the country school inspection offers little or no inducement for a man to stay more than a year or two at it. Such work cannot always lead to public health work proper, and there is ever present the risk of being side-tracked and of remaining a mere officer of the Education Authority without prospects of promotion or advance in salary beyond £300 or £350 per annum. With the advent of the tuberculosis officer the school medical officer became even more discouraged, for he saw men who had been lucky enough to spend a few months in a sanatorium starting off on a none too arduous job at salaries of £500 a year, and even junior men starting at £400, with motor-cars and other emoluments. It is not too much to say that an efficient medical examiner of school children requires a wider clinical knowledge than that necessary for a one-disease man such as the tuberculosis officer often is, while the amount of good which a well-conducted scheme of school inspection can do for the nation at large is quite as urgent and more free from controversy than the prevention of consumption plans now so numerous. As the result of the present state of the School Medical Service changes among the officers are becoming increasingly frequent. For those who find the work monotonous and dull it is well that they should seek other fields, but meanwhile the comparative value of the statistics becomes greatly diminished by the continual insertion of a different personal factor.

### THE NEW PROFESSORSHIP AT EDINBURGH.

The reorganisation of clinical teaching at Edinburgh University is being gradually accomplished; and undoubtedly reforms of the old system were genuinely necessary. The latest instalment is the appointment of Dr. William Russell, M.D., F.R.C.P. Edin., to the new Moncrieff Arnott Chair of Clinical Medicine. Dr. Russell is at present physician and lecturer on clinical medicine in the Royal Infirmary, Edinburgh, and has been for many years a lecturer on the practice of medicine in the Edinburgh School of Medicine. Among other distinctions in a long and honourable career he has been editor of the *Scottish Medical and Surgical Journal*. His published writings have been almost exclusively concerned in the diseases of the heart and circulatory system. He is an Edinburgh man, and qualified as long ago as 1876. He is sixty-one years of age, so that his appointment to a brand-new professorship, involving hard work in novel conditions, is a testimony to the electors' confidence in his youthfulness of mind. To those at a distance it reads like a slightly hazardous experiment; but the Scots are a hardy race, and know their own business better than English folk can teach it them.

### THIS WEEK'S DRUG MARKET.

An improvement in business cannot be long delayed in view of the near approach of the winter season, and the probability is that the remainder of the present year will be busier than the corresponding period last year, when the medical benefit under the Insurance Act was not in operation. In the meantime the Drug market continues quiet with few price changes either in an upward or a downward direction. There has been no improvement in the demand for cod-liver oil, and the market cannot be described as firm. Castor oil is rather lower in price. Essence of lemon is cheaper. Menthol still has a higher tendency. Opium is quiet and unchanged in price, but morphine is just a shade lower. Senega tends towards higher prices. Podophyllum is rather dearer. The price of carbolic acid is inclined downwards. There has been no further development in the Quinine market. Orris root is rather dearer, and it seems likely that prices will further advance.

